# Relative Vaccine Effectiveness of the Third Dose of CoronaVac or BNT162b2 Following a Two-Dose CoronaVac Regimen: A Prospective Observational Cohort Study from an Adult Vaccine Center in Turkey

**DOI:** 10.3390/vaccines10071140

**Published:** 2022-07-18

**Authors:** Meliha Cagla Sonmezer, Gulcin Telli Dizman, Enes Erul, Taha Koray Sahin, Tuğba Saricaoglu, Alparslan Alp, Mine Durusu Tanriover, Omrum Uzun, Serhat Unal, Murat Akova

**Affiliations:** 1 Infectious Diseases and Clinical Microbiology Department, Hacettepe University Faculty of Medicine, 06100 Ankara, Turkey; gulcin.telli@hacettepe.edu.tr (G.T.D.); tugbasaricaoglu@hacettepe.edu.tr (T.S.); ouzun@hacettepe.edu.tr (O.U.); sunal@hacettepe.edu.tr (S.U.); makova@hacettepe.edu.tr (M.A.); 2 Internal Medicine Department, Hacettepe University Faculty of Medicine, 06100 Ankara, Turkey; eneserul@hacettepe.edu.tr (E.E.); koraysahin@hacettepe.edu.tr (T.K.S.); mdurusu@hacettepe.edu.tr (M.D.T.); 3 Microbiology and Clinical Microbiology Department, Hacettepe University Faculty of Medicine, 06100 Ankara, Turkey; aalp@hacettepe.edu.tr

**Keywords:** COVID-19 vaccine, booster dose, third dose, vaccine effectiveness

## Abstract

Coronavirus disease 2019 (COVID-19) continues to pose a threat to public health with the potential for the emergence of new variants. Vaccines are the milestones to control and slow down the damage of the pandemic. As of January 2021, a two-dose regimen with CoronaVac was authorized in Turkey. Due to the waning seroprevalence rate of SARS-CoV-2 over time, BNT162b2 or CoronaVac has been administered as the third dose following a two-dose CoronaVac regimen as a national vaccination policy. As of 14 January 2021, 5243 volunteers who received two doses of the CoronaVac vaccine at Hacettepe University Adult Vaccine Center were followed prospectively. In our study, relative vaccine effectiveness (VE^ff^) for the third dose of the CoronaVac was 58.24% and 87.27% for BNT162b2 in preventing symptomatic COVID-19 cases. There were no hospitalizations, intensive care unit admissions, or deaths in third-dose booster groups with either BNT162b2 or CoronaVac, yielding 100% effectiveness. Both homologous or heterologous third-dose boosters provided further protection against severe COVID-19 and should be prioritized as an effective strategy to combat the COVID-19 pandemic.

## 1. Introduction

Coronavirus disease 2019 (COVID-19) has affected the lives of millions for more than two years, with successive peaks led by mutations and new variants [1,2,3]. Globally, as of 17 May 2022, there have been 519.729.804 confirmed cases of COVID-19, and 6,268,281 deaths. Vaccination against SARS-CoV-2 has been among the most critical strategies to curb and change the course of the COVID-19 pandemic worldwide; and as of 14 May 2022, a total of 11,660,363,722 vaccine doses have been administered [4].

The Ministry of Health of Turkey approved a two-dose regimen of the CoronaVac, an inactivated COVID-19 vaccine developed by the Chinese biopharmaceutical company Sinovac Life Sciences (Beijing, China), for emergency use against severe acute respiratory syndrome coronavirus 2 (SARS-CoV-2) and began mass vaccination on 13 January 2021. 

As of 17 October 2021, CoronaVac has been approved in 41 additional countries for emergency use [5]. More than 750 million doses have been administered worldwide [5]. Due to the need for COVID-19 vaccines, data on the efficacy of vaccines in clinical trials were scarce, and there was concern about vaccine effectiveness [6,7,8]. 

Because of waning immunity over time and against new variants, the third-dose booster was recommended with either CoronaVac or mRNA (BioNTech/Fosun Pharma; BNT162b2) vaccines in early July 2021. We aimed to evaluate the relative VE^f^^f^ and additional protection of third doses of BNT162b2 or CoronaVac against mild/moderate infections, severe/fatal illnesses, and mortality in this real-life observational study in Turkey. We believe a detailed analysis of VE^ff^ will help policy makers manage the booster dose strategy after two doses of CoronaVac vaccination and reduce vaccine hesitancy in the community by increasing public confidence in vaccination programs.

## 2. Materials and Methods

### 2.1. Study Design, Study Setting, and Participants

This study was conducted at an adult vaccination center of a tertiary care university hospital in Turkey as an observational study. The Strengthening the Reporting of Observational Studies (STROBE) in Epidemiology guidelines [9] were applied, and the STROBE checklist was followed in this study: COVID-19 vaccines require approximately two weeks to be effective; a person is not fully vaccinated until two weeks after receiving the recommended number of doses for the vaccine. A person who tests positive for COVID-19 between the first and second doses of two-dose vaccines or tests positive before two weeks following their last dose is not considered a breakthrough case. We evaluated the CoronaVac VE^ff^ with the relationship between COVID-19 events and outcomes 14 days after complete dose administration. Lactation, pregnancy, recent (within the last six months) or planned use of immunosuppressive therapy, known allergy to components of the study vaccine or placebo, or use of immunoglobulins or any blood products within the previous three months, asplenia, and any confirmed or suspected immunodeficiency disease (n = 335) were among the exclusion criteria.

**Figure 1:**
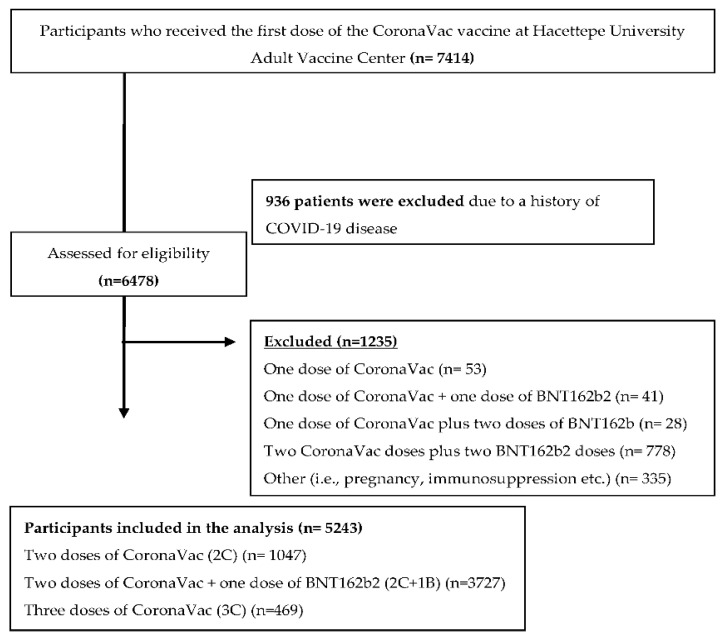
Flow diagram of the patient selection process.

Figure 1 illustrates the flow diagram of patient enrollment. Participants were stratified into three groups according to vaccination status, such as two doses of CoronaVac (2C) or two doses of CoronaVac plus one BNT162b2 (2C + 1B) dose or three doses of CoronaVac (3C). The first dose of the CoronaVac vaccine was administered in our adult vaccination center on 14 January 2021. In this study, adults over 18 who received the first dose of CoronaVac vaccine as of 14 January 2021 in our center were followed up prospectively for COVID-19 infection until 10 January 2022, thus potentially excluding the era with any of the Omicron variants.

### 2.2. Data Collection Procedures

We used the prospective observational cohort dataset of our adult vaccination center to provide estimates of the effectiveness of the CoronaVac vaccine in preventing COVID-19 and related hospitalizations, admission to the intensive care unit (ICU), and death. We calculated VE^ff^ estimates among different age groups. The follow-up of the participants for subsequent COVID-19 infection was conducted via the public health management system of the Turkish Ministry of Health. Reverse transcriptase–polymerase-chain-reaction (RT-PCR) assays have been performed with kits provided nationwide by the Ministry of Health. Viral nucleic acid isolation from the samples was achieved using Bio-Speedy vNAT viral nucleic acid buffer (Bioeksen R&D Technologies Ltd., Sarıyer, Turkey). The Bio-Speedy SARS-CoV-2 Emerging Plus real-time PCR kit (Bioeksen R&D Technologies Ltd., Sarıyer, Turkey) detected SARS-CoV-2 in respiratory samples. The kit is a one-step reverse transcription and multiplex real-time PCR test intended for the qualitative detection of RNA from the SARS-CoV-2 and the differentiation of the B.1.1.7 and the variants containing the E484K and L452R mutations in nasopharyngeal swabs, oropharyngeal swabs, nasal swabs, nasopharyngeal aspirates, saliva and bronchoalveolar lavage samples from individuals suspected of COVID-19. 

The primary outcome was the incidence of symptomatic COVID-19 cases validated by RT-PCR, the number of hospitalizations or deaths at least 14 days after receiving the last (second or third) vaccine dose.

### 2.3. Data Analysis

Statistical analyses were performed using the SPSS software version 18. A type-I error of less than 5% was interpreted as statistically significant. Descriptive statistics were expressed as numbers and percentages for categorical variables and as the mean, standard deviation, median, and minimum–maximum for numerical variables. Kaplan–Meier curves were used to estimate the cumulative incidence of SARS-CoV-2 infection in the booster group and the non-booster group. The log-rank test compares COVID-19 event time among the gender and age groups. Risk ratios and risk differences were calculated by dividing and subtracting the period-specific risk estimates, respectively. Vaccine effectiveness was defined as 1 minus the risk ratio, and 95% confidence intervals were estimated using the non-parametric percentile bootstrap method with 500 repetitions.

## 3. Results

A total of 7414 volunteers who received the first dose of the CoronaVac vaccine at Hacettepe University Adult Vaccine Center were included in this study. Patients with a history of COVID-19 infection (n = 936) were excluded (Figure 1).

The vaccination status of the participants included a broad scheme of heterologous vaccines of CoronaVac and BNT162b2 vaccines in our study. Since the VE^ff^ of the third dose with CoronaVac or BNT162b2 as a booster was compared against two doses of CoronaVac, participants who received one dose of CoronaVac, one dose of CoronaVac plus one dose of BNT162b2, one dose of CoronaVac plus two doses of BNT162b, and two CoronaVac doses plus two BNT162b2 doses were also excluded in this study. All participants were at least vaccinated with two CoronaVac doses. A total of 5243 participants were followed prospectively for developing COVID-19 infection until 10 January 2022. The follow-up time of participants varied from 66 to 362 days. The median age of participants was 40 years (min. 18–max. 99), with 2636 (50.2% of the total) being over 40 years of age. A total of 2821 (53.8%) participants were female, and 2422 (46.2%) were male. Approximately half of the participants (n = 2673, 50.9%) were health care workers. The vast majority of participants (71%, n = 3727) were vaccinated with two CoronaVac doses plus one BNT162b2 dose (2C + 1B), followed by 19.96% (n = 1047) vaccinated with two CoronaVac doses (2C), and 8.9% (n = 469) with three doses of CoronaVac vaccine. The baseline characteristics of the participants are shown in Table 1.

From at least 14 days after the second CoronaVac vaccine, a total of 366 (6.9%) cases with COVID-19 infection were identified among 5243 participants. Forty-one (11.2%) required hospitalization and 15 (4.1%) died (Table 2).

**Figure 2:**
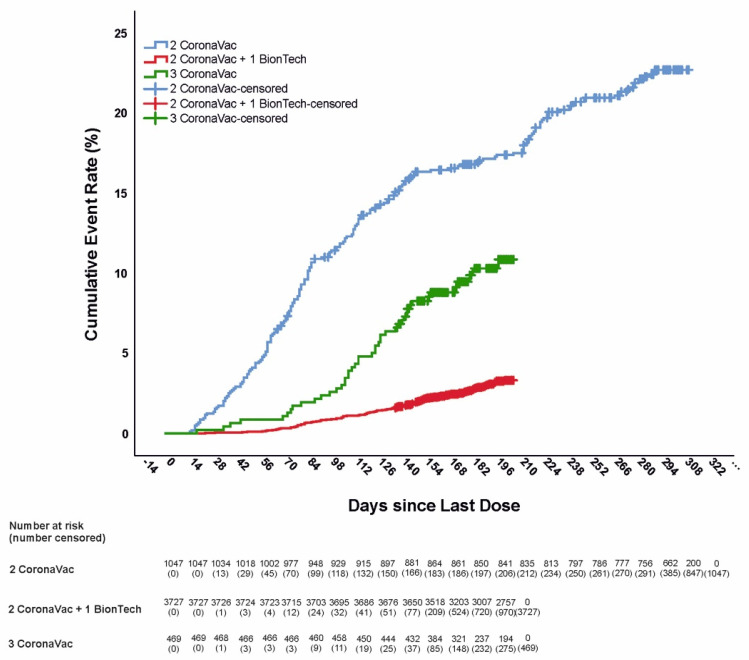
Cumulative incidence of SARS-CoV-2 infection categorized by vaccination status assessed from 2 weeks after receipt of the last dose of vaccine.

Cumulative incidences of documented COVID-19 infections in the three vaccination groups are shown in Figure 2. More than half of these cases (56%, n = 208) had received only two doses of CoronaVac. Hospitalizations and deaths occurred only in the two-dose group (*p* < 0.001) (Table 2).

The estimated VE^ff^ from 2 weeks after receipt of the last dose of vaccine was 87.27% (95% CI 84.21–89.74; incidence rate 57.62 per 1 000 person-years) for preventing PCR-confirmed symptomatic COVID-19 infection in two CoronaVac doses plus one BNT162b2 dose group compared to two doses of CoronaVac. VE^ff^ was 58.24% (95% CI 43.43.–69.17; incidence rate 192.58 per 1 000 person-years) for preventing PCR-confirmed symptomatic COVID-19 infection in three CoronaVac doses group compared to two doses of CoronaVac (Table 3). Vaccination with third doses as a booster dose with CoronaVac or BNT162b2 vaccine demonstrated complete effectiveness for preventing COVID-19-related hospitalization (100%) (*p* < 0.001) (Table 2). 

CoronaVac and BNT162b2 booster were both safe and well-tolerated in patients with COVID-19. Although 10/44 (22.7%) and 50/114 (43.8%) of CoronaVac and BNT162b2 booster vaccines, respectively, presented some adverse events, the majority of adverse events were mild or moderate and transient. The most common vaccine reactions were headache and injection site pain. Diarrhea (%9) was more common for CoronaVac booster, whereas fever, myalgia, and malaise were commonly reported for BNT162b2 booster (28%, 35%, and 30.7%). There were safety concerns emerging as grade 2 liver transaminases elevations only for two patients vaccinated with two CoronaVac doses plus one BNT162b2 dose over a month. There were no identifiable patterns of frequency observed for adverse events according to the booster vaccine type or age group; adverse events were most likely to occur in the first few days following the booster dose. Our findings were similar to the results of others [10,11].

## 4. Discussion

Vaccination with CoronaVac is used primarily in countries with a shortage of mRNA vaccines since the BNT162b2 vaccine requires cold chain infrastructure to maintain efficacy [12,13]. The main concern with inactivated vaccines is the production of lower levels of neutralizing antibodies compared to the BNT162b2 vaccine; thus, more breakthrough infections are anticipated [14]. In addition, humoral immunity attained with two doses of CoronaVac decreases considerably in 3–6 months [15,16,17]. Therefore, questions and concerns remain about how effective CoronaVac is as a third-dose booster vaccine in preventing infection, hospitalization, and death compared to the BNT162b2 vaccine. 

The first interim phase 3 trial data for CoronaVac in Turkey included 10,214 participants, with almost 2/3 assigned to the vaccine and 1/3 given to placebo [9,18]. The VE^ff^ was found to be 83.5% to prevent symptomatic infection among fully vaccinated people. Notably, because of the modest sample size in this analysis, only a few COVID-19 cases were observed (41 in total, with 9 in the vaccine group and 32 in the placebo group). A national cohort study from Chile with a more extensive dataset included 10.2 million people covering three months from 2 February to 1 May 2021, thus potentially excluding the era with any of the two Omicron subvariants. More than 4 million individuals received two doses of CoronaVac vaccine with a subsequent COVID-19 infection in more than 218,000 in the cohort, resulting in over 22,000 hospitalizations and 4000 deaths. Among those fully vaccinated with CoronaVac, it was estimated that the vaccine was 65.9% effective for preventing SARS-CoV-2 infection, 85.3% effective for preventing hospitalization, and 86.5% effective for preventing death from SARS-CoV-2 infection [19]. Since the variants are different at the time of these studies, it may partly explain differences in estimates of VE^ff^ between Turkey and Chile [18,19].

In Brazil, the VE^ff^ of CoronaVac against Gamma variant was 46.8% to prevent SARS-CoV-2 infection, 55.5% for hospitalization, and 61.2% against death. The VE^ff^ against all outcomes dropped with older age and mutations [20]. Another study demonstrated that neutralizing antibody titers induced by two doses of CoronaVac declined to near or below the lower limit of seropositivity after six months. However, a third dose given eight months after the second dose led to a substantial boost in immune response [21]. Bochnia et al. found that CoronaVac’s antibody levels peaked 40 days after the first dose and steadily declined over time, dropping in half about one to two months [22] seropositivity rate remained at nearly 95% among recipients of the BNT162b2 vaccine [23]. Although the complexity and variety of the immune system beyond neutralizing antibodies, it has been shown that neutralizing antibody levels are highly predictive of resistant protection from symptomatic SARS-CoV-2 infection [14]. 

Recently, in a study, Keskin et al. compared health care workers’ spike antibody (IgG-S) response to a third dose of CoronaVac or the BNT162b2 following a two-dose CoronaVac regimen. They found that CoronaVac or BNT162b2 as a booster increased IgG-S levels [24]. Similarly, serum human neutralizing antibodies increased and showed great effectiveness against variants including delta after a third-dose booster of inactivated SARS-CoV-2 vaccine [25]. Additionally, observational studies showed BNT162b2 and CoronaVac as a booster in people who had already been vaccinated with CoronaVac and found that BNT162b2 provided a significantly higher increase in humoral response when compared to a homologous booster [24,26]. 

As a clinical reflection of laboratory studies, our study has shown that two doses of CoronaVac vaccines are more effective when boosted with a single mRNA vaccine than three doses of CoronaVac. However, the relative VE of three doses of CoronaVac was above 50%, which meets the critical or minimal effectiveness criteria of vaccines for indicating pandemic usage as a target by WHO. Our findings revealed that CoronaVac and BNT162b2 vaccines as third dose have high effectiveness against symptomatic SARS-CoV-2 infection and severe COVID-19 infection with the emergence of viral variants. The three-dose groups observed no severe cases requiring hospitalization, ICU admission, and death. As previously mentioned in the studies, the primary mechanism that supports our findings is that an inactivated boost dose of the SARS-CoV-2 vaccine may result in a complex interaction of immunity with T cell proliferation and increased humoral immunity [27]. It is suggested that immune memory remains intact for the recall response to homolog vaccine stimulation [27]. The prevalence of adverse effects was similar across the CoronaVac and BNT162b2 booster groups. Although there have been few studies evaluating vaccine effectiveness caused by heterologous COVID-19 booster vaccinations, the currently available data indicate that getting a booster dose of a different vaccine had an acceptable safety profile and was just as protective as getting another dose of the same vaccine as the initial series.

Our study is among the first assessing the results of mRNA or CoronaVac vaccine as a third-dose booster in a real clinical setting, providing novel evidence to the limited data in the literature. Among the main strengths of our study, we have followed up with participants prospectively for at least two months to one year. Secondly, with its high number of COVID-19 cases, Turkey offers a unique environment to evaluate whether a booster dose should be given and the relative VE^ff^ of the third dose of CoronaVac or BNT162b2 since the first two doses mainly were authorized with CoronaVac. Thirdly, we calculated VE^ff^ in compliance with the most recent WHO recommendation for evaluating the effectiveness of the COVID-19 vaccination [28]. There are several limitations in our study that should be mentioned. The study design is susceptible to selection bias and confounding factors such as age, sex, and underlying comorbidities that may alter vaccination efficacy. Secondly, we could not exclude the impact of programmatic concerns, including a defective cold chain, long gaps between doses, and imperfect vaccination schedules on vaccine effectiveness. Comparing the effectiveness of various vaccines should be done with caution because the median length between the second dose and booster dose may vary significantly between doses. Among the main limitations of this study is that we could not take into account factors such as the time period since the last dosage of the vaccine. Therefore, COVID-19 incidence and exposure time may vary between periods. We should also bear in mind that infection risk varies throughout time, as does the probability of accessibility of vaccination, and a previous asymptomatic infection might occur in the study population. In our study, unfortunately, the spike and non-neutralizing viral antibody levels of participants were not available. Nonetheless, the FDA does not advise antibody testing to evaluate immunity or protection from COVID-19, particularly among those who had vaccination [29]. Finally, our data exclude vaccine effectiveness against the Omicron variant and may not be generalized to the current status of the pandemic.

## 5. Conclusions

We found that cumulative incidences of COVID-19-related events were significantly lower in the participants with third doses as a booster dose with CoronaVac or BNT162b2 vaccine than in participants with two CoronaVac doses. Third doses of BNT162b2 or CoronaVac provide significant further protection against severe COVID-19 and should be prioritized as an effective strategy; the world requires every dosage of any safe and effective SARS-CoV-2 vaccine available for booster doses to reduce hospitalization and death. 

## Figures and Tables

**Table 1:** Characteristics of study cohort categorized by vaccination status.

	Total Number of Fully Vaccinated Persons No. (%)	2C No. (%)	2C + 1B No. (%)	3C No. (%)	*p*-Value
**Total**	5243	1047 (19.96)	3727(71)	469 (8.9)	
**Sex, no. (%)**					
Male	2422 (46.2)	535 (51.1)	1651 (44.3)	236 (50.3)	<0.001
Female	2821 (53.8)	512 (48.9)	2076 (55.7)	233 (49.7)	
**Age**					
Median age (yrs) ± SD Mean (min.–max.)	43 ± 17 40 (18–99)	42 ± 17 37 (18–99)	42 ± 16 39 (19–95)	49 ± 18 45 (21–92)	<0.001
**Age group, no. (%)**					
18–29 yrs	1414 (27)	265 (25.3)	1091 (29.3)	58 (12.4)	<0.001 <0.001
30–39 yrs	1193 (22.8)	313 (29.9)	782 (21)	98 (20.9)	<0.001
40–49 yrs	1119 (21.3)	203 (19.4)	791 (21.2)	125 (26.7)	0.006
50–59 yrs	581 (11.1)	103 (9.8)	413 (11.1)	65 (13.9)	0.07
60–69 yrs	318 (6.1)	39 (3.7)	260 (7)	19 (4.1)	<0.001
70–79 yrs	384 (7.3)	62 (5.9)	262 (7)	60 (12.8)	<0.001
80–89 yrs	212 (4)	54 (5.2)	117 (3.1)	41 (8.7)	<0.001
90–99 yrs	22 (0.4)	8 (0.8)	11 (0.3)	3 (0.6)	0.086
**Health care worker,** **no. (%)**	2673 (51)	583 (55.7)	1890 (50.7)	200 (42.6)	<0.001
**Persons with COVID-19, no. (%)**	366 (7)	208 (19.9)	114 (3.1)	44 (9.4)	<0.001

Abbreviations: 2C: two doses of CoronaVac; 2C + 1B: 2 CoronaVac + 1 BNT162b2; 3C: three doses of CoronaVac; SD: standard deviation.

**Table 2:** Clinical status of patients with COVID-19 by vaccination status.

Clinical Status	Persons with COVID-19 (n = 366)	2C (n = 208)	2C + 1B (n = 114)	3C (n = 44)	*p*
Outpatient	325 (88.8)	167 (80.3)	114 (100)	44 (100)	<0.001
Hospitalized—no oxygen therapy	16 (4.4)	16 (7.7)	0 (0)	0 (0)	<0.001
Hospitalized—oxygen therapy	10 (2.7)	10 (4.8)	0 (0)	0 (0)	<0.001
Death	15 (4.1)	15 (7.2)	0 (0)	0 (0)	<0.001

Abbreviations: 2C: two doses of CoronaVac; 2C + 1B: 2 CoronaVac + 1 BNT162b2; 3C: three doses of CoronaVac.

**Table 3:** The effectiveness of the third dose of CoronaVac or BNT162b2 following a two-dose CoronaVac regimen in preventing COVID-19 cases after 14 days from second or third dose.

Vaccination Status	Person-Days (Total Number)	Person-Days (Median-(IQR))	SARS-CoV-2 Infections (n)	Incidence (1.000 Person-Years)	Incidence Rate (%)	Vaccine Effectiveness (Percent (95%CI))
**2C**	269,172	303 (255–307)	208	282,050	0.77	-
**2C + 1B**	722,121	202 (195–206)	114	57,622	0.15	87.27 (84.21–89.74)
**3C**	83,390	182 (160–201)	44	192,589	0.52	58.24 (43.43–69.17)

Abbreviations: 2C: two doses of CoronaVac; 2C+1B: 2 CoronaVac + 1 BNT162b2; 3C: three doses of CoronaVac.

## Data Availability

The data supporting this study’s findings are available on request from the corresponding author. The data are not publicly available due to privacy or ethical restrictions.
